# RNA Combined with Nanoformulation to Advance Therapeutic Technologies

**DOI:** 10.3390/ph16121634

**Published:** 2023-11-21

**Authors:** Eduarda Santos Lima, Déborah dos Santos, Atena Liriel Souza, Maria Eduarda Macedo, Mariana Evangelista Bandeira, Sérgio Santos Silva Junior, Bianca Sampaio Dotto Fiuza, Vinicius Pinto Costa Rocha, Larissa Moraes dos Santos Fonseca, Danielle Devequi Gomes Nunes, Katharine Valéria Saraiva Hodel, Bruna Aparecida Souza Machado

**Affiliations:** SENAI Institute of Innovation (ISI) in Health Advanced Systems (CIMATEC ISI SAS), University Center SENAI/CIMATEC (Integrated Manufacturing and Technology Campus), Salvador 41650-010, Brazil; eduardalimasha@gmail.com (E.S.L.); deborah.dsantos15@gmail.com (D.d.S.); atenalsouza@outlook.com (A.L.S.); mariaefmw@gmail.com (M.E.M.); mari.bandeira89@gmail.com (M.E.B.); jsilva.ss546@gmail.com (S.S.S.J.); bianca.fiuza@fbter.org.br (B.S.D.F.); vinicius.rocha@fieb.org.br (V.P.C.R.); larissa.fonseca@fieb.org.br (L.M.d.S.F.); danielle.nunes@fieb.org.br (D.D.G.N.); katharine.hodel@fieb.org.br (K.V.S.H.)

**Keywords:** RNA replicon therapy, RNA replicon vaccines, self-amplifying RNA

## Abstract

Nucleic acid-based therapies have the potential to address numerous diseases that pose significant challenges to more traditional methods. RNA-based therapies have emerged as a promising avenue, utilizing nanoformulation treatments to target a range of pathologies. Nanoformulation offers several advantages compared to other treatment modalities, including targeted delivery, low toxicity, and bioactivity suitable for drug loading. At present, various types of nanoformulations are available, such as liposomes, polymeric nanoparticles (NPs), magnetic NPs, nanoshells, and solid lipid nanoparticles (SLNs). RNA-based therapy utilizes intracellular gene nanoparticles with messenger RNA (mRNA) emerging prominently in cancer therapy and immunotechnology against infectious diseases. The approval of mRNA-based technology opens doors for future technological advancements, particularly self-amplifying replicon RNA (repRNA). RepRNA is a novel platform in gene therapy, comprising viral RNA with a unique molecular property that enables the amplification of all encoded genetic information countless times. As a result, repRNA-based therapies have achieved significant levels of gene expression. In this context, the primary objective of this study is to furnish a comprehensive review of repRNA and its applications in nanoformulation treatments, with a specific focus on encapsulated nanoparticles. The overarching goal is to provide an extensive overview of the use of repRNA in conjunction with nanoformulations across a range of treatments and therapies.

## 1. Introduction

While drug development has made significant progress over the years, there remains a pressing need for further advancements in this field [1]. Technological innovations and genomic research have opened numerous treatment possibilities [2]. Moreover, nucleic acid-based therapies hold the potential to address or even cure many diseases that have proven resistant to traditional treatment methods [2]. The discovery of small interfering RNA (siRNA) and antisense RNA, which effectively suppress the expression of specific genes in cells affected by viruses or cancer, has sparked increased interest in RNA treatments [3]. These advances encompass a range of mechanisms, including approaches such as inhibiting messenger RNA (mRNA) translation, gene expression interference via siRNA, self-amplifying replicon RNA (repRNA), catalytically active ribozymes, and protein-binding RNA molecules. Consequently, RNA-based therapies find applications in diagnostics and certain treatment modalities [4].

Synthetic mRNA serves as a versatile tool for producing proteins and peptides, finding extensive use in pharmaceutical applications, including cancer immunotherapy [5]. It is generated in a cell-free system through in vitro transcription (IVT) from a DNA template [6]. Acting as a natural ligand for various receptors, exogenous mRNA stimulates the release of type I interferon and pro-inflammatory cytokines, providing it with inherent adjuvanticity [5] On the other hand, repRNA, with sizes ranging from 12 to 15 kilobases, stem from viral genomes lacking at least one structural protein gene, enabling sustained antigen production without the risk of producing infectious progeny [7]. However, their susceptibility to RNase and inefficient uptake by dendritic cells represent key limitations. Compared to conventional mRNA, repRNA triggers prolonged gene expression lasting weeks to months after a single injection, making it a promising candidate for sustained antigen expression in vaccine design [8]. repRNA vaccines have emerged as promising candidates, showcasing exemplary safety and immunogenicity, and have already demonstrated encouraging results in various clinical trials [9,10].

Regarding siRNA, its mechanism relies on post-transcriptional gene silencing, known for its specificity in targeting disease-related genes [11]. However, siRNAs face challenges such as low cellular uptake and susceptibility to degradation, necessitating protective carriers for efficient delivery into target cells [12,13]. Various delivery systems, including nanocarriers, aptamers, peptides, sugars, proteins, and antibodies, have been developed to address these limitations [12,13]. In mammalian cells, siRNAs play a crucial role in RNA interference (RNAi), with minimal concentrations as low as 2000 siRNAs per cell being sufficient for effective gene knockdown [14]. Compared to DNA, RNA has several distinctive qualities that make it a potent biomaterial. Notably, RNA is capable of folding into several tertiary structures with unique activities [3]. The effectiveness of RNA therapy is significantly enhanced by its synergistic association with encapsulated nanoparticles, which facilitate targeted and controlled delivery, thereby increasing its therapeutic potential.

RNA nanotechnology offers several advantages for therapeutic applications. These advantages encompass passive targeting due to its nano-scale size and branched structure, precise synthesis control, limited nonspecific cell membrane crossing, high water solubility, and reduced immunogenicity. Additionally, RNA nanotechnology provides multivalency for module conjugation, favorable in vivo profiles, and specific delivery potential [15]. Furthermore, RNA’s classification as a chemical reagent implies more favorable regulatory processes compared to protein-based clinical reagents.

It is important to note that lipid-encapsulated nanoparticles containing small interfering double-stranded RNA (siRNA) have been employed to target transthyretin (TTR) mRNA, leading to the degradation of TTR deposits in patients with TTR-mediated hereditary amyloidosis [9]. This RNA interference drug, Patisiran (ONPATTRO™), has received approval in both the US and Europe for single intravenous infusion [16]. In recent years, mRNAs generated through IVT have gained prominence as promising candidates for the development of new drugs and vaccines [1,16].

Despite considerable progress in RNA nanotechnology for medical applications, several challenges persist. These include RNA’s chemical instability, the need for thermodynamic stability in RNA nanoparticles, construction costs, the requirement for favorable pharmacological profiles, specific cell targeting, and the prevention of degradation in the endocytic pathway [3]. Overcoming these challenges is crucial for realizing the full potential of RNA nanotechnology in therapeutics.

Consequently, intensive research efforts are currently underway in the fields of cancer immunotherapy, vaccine development for infectious diseases, gene editing, and protein replacement, with the goal of advancing our understanding in this rapidly evolving field [9] In light of these developments, this study seeks to provide a comprehensive review of RNA delivery systems, focusing particularly on encapsulated nanoparticles. The primary objective is to examine the significant levels of gene expression achieved through repRNA-based therapies within the broader context of nanoformulation treatments. The overarching goal is to offer an extensive overview of repRNA’s utilization in conjunction with nanoformulations, encompassing a range of treatments and therapies.

## 2. Nanotechnology’s Impact on Healthcare: Advancements, Applications, and RNA Nanotechnology

The prefix “nano”, derived from Greek, signifies something minuscule, measuring as small as less than one-millionth of a meter [17]. This minuscule scale is fundamental to nanotechnology’s core concept. Conversely, technology denotes the systematic study of processes within a specific domain [18]. However, nanotechnology takes this a step further. Following this initial definition, nanotechnology is regarded as a scientific discipline dedicated to comprehending matter at nanometric scales, typically ranging from 1 to 100 nanometers, with the aim of precise control and manipulation to usher in technological advancements across diverse domains [19].

Our civilization has greatly benefited from nanotechnology, which has made significant contributions to numerous prominent industries [20]. Among these industries, over the past two decades, nanotechnology’s advancement in the medical and healthcare sectors have played a crucial role in preventing, diagnosing, and treating various illnesses [20].

Within the realm of healthcare, the application of nanotechnology is termed “nanomedicine,” a field that has yielded a profound understanding of cellular mechanisms in living organisms [21]. This profound understanding has paved the way for various applications encompassing pharmaceutical therapy and the prevention, diagnosis, monitoring, and treatment of many diseases [22].

The implications of nanotechnology in scientific research are highly promising, transcending the limitations of conventional therapies by enhancing the mechanisms employed, particularly in safeguarding the desired drug [21]. To achieve these advancements, nanotechnology employs top-down and bottom-up assembly methods to create and utilize materials at the nanometer scale. Notably, DNA, RNA, and protein macromolecules possess inherent properties at the nanoscale, making them promising building blocks for the bottom-up construction of nanostructures and nanodevices [15].

Research into the folding and structure of RNA has a long history. However, it is important to note that RNA nanotechnology distinguishes itself from conventional RNA structure and folding studies [23]. RNA nanotechnology is the study of RNA-based structures with a primary framework at the nanoscale [24]. This unique focus necessitates a comprehensive understanding of not only intramolecular interactions and folding but also intermolecular interactions. A crucial aspect of RNA nanotechnology involves characterizing the physical, chemical, biological, and pharmacological properties of nanoparticles that researchers can homogenize through purification [23]. An important milestone in the field was the assembly of RNA dimer, trimer, and hexamer nanoparticles using reengineered RNA fragments derived from pRNA (packaging RNA), a vital component responsible for driving the DNA packaging motor in bacteriophage phi29 [25]. 

RNA nanotechnology, as opposed to conventional RNA biology research, focuses on using the characteristics of RNA to create structures for use in nanomedicine [24]. This approach is particularly promising for therapeutic applications, where nanoformulations offer synergistic actions and efficient delivery systems, notably in the treatment of diverse cancer types [26]. Beyond medicinal technology, a range of industrial sectors, including electronics, environmental science, food production, and textiles, have expressed keen interest in nanotechnology due to its potential to enhance product quality, safety, and durability [22].

### 2.1. Nanoformulation

Researchers engineer nanoformulations to optimize drug delivery, increase drug solubility, improve bioavailability, and enable targeted drug release, thereby enhancing the overall efficacy and safety of pharmaceuticals [26]. In terms of therapeutic effectiveness, researchers have observed that nanoparticles exhibit optimal internalization by cancer cells, especially in the treatment of diseases like breast cancer [27]. Their enhanced permeation into the target cells not only improves drug delivery, but also renders the nanoformulation more cytotoxic when compared to free drugs [28].

These nanodelivery technologies can also apply to phytocompounds. Various phytocompounds, including cannabidiol (CBD), have received attention for delivery using nanocarriers as a viable platform, with the aim of restricting the variety of negative consequences [29]. Nanodelivery technologies have improved the stability of phytocompounds, enhanced their absorption, shielded them from early enzymatic depletion or metabolism within the body, and prolonged their circulation duration [29].

Therefore, the significance of this technology lies in advancing the treatment of various diseases and offering expanded therapeutic possibilities [30]. Furthermore, its economic advantages are compelling, as it allows for easy industrial scale-up, making it a practical choice in this sector. These innovative nanoformulations hold the potential to revolutionize drug delivery and substantially improve patient outcomes across a wide range of medical conditions. With this in mind, Table 1 shows some nanoformulations and their main components that have already been approved for therapeutic use by regulatory agencies such as the FDA.

#### 2.1.1. Liposomes

Nanoparticles serve as drug carriers, and one of the key applications of this nanotechnology is in disease therapy [31]. An excellent example is liposomes, which, as the name suggests, are lipid-based nanoparticles that are usually 50–500 nanometers in diameter size [32,33]. They consist of phospholipid and cholesterol bilayers, with their number varying depending on the classification and determined by their size and the number of lamellae [34].

Liposome vesicles are spherical and spontaneously form in aqueous media, offering versatility in terms of the drug and the route of administration [35]. The administered drug may be either polar (water-soluble), concentrating in the internal aqueous phase, or apolar (fat-soluble), containing compounds with this characteristic [36]. The variation in bilayers is crucial for their function, as these structures are essential for encapsulating drugs with different sizes and solubilities [37]. Furthermore, the choice of a specific liposome classification influences its targeting specificity towards organs, cells, and tissues [38].

The use of liposomes offers numerous advantages. Liposomes, with their unique properties, serve as flexible and adaptable nanocarriers for drug delivery [39]. Their amphiphilic nature enables the encapsulation of both hydrophilic and hydrophobic medications, enhancing their versatility, providing excellent biocompatibility and biodegradability, along with the isolation of the drug from the surrounding medium [39,40]. By preventing direct contact with healthy cells, liposomes can reduce the toxicity of chemotherapeutic drugs, while also enhancing the stability and bioavailability of these compounds [39]. Drug concentrations at sites of action are seven to nine times greater when employing drug-loaded liposomes as delivery vehicles than when using medicines alone without carriers [41]. However, there are still disadvantages to consider, such as their relatively large particle size and rapid clearance by the immune system [42].

Nevertheless, the applications of liposomes are extensive. They find use in protecting drugs from degradation in vivo, directing drug delivery to the desired site, controlling drug release, modifying and refining biodistribution, and increasing bioavailability and solubility [43]. As the first nano-delivery system, liposomes have achieved considerable clinical success, with well-consolidated and established pharmacokinetics and delivery pathways [16]. Consequently, liposomes have demonstrated high compatibility with RNA for complexed use in cancer immunotherapies [44]. These adaptable lipid-based nanoparticles continue to be a foundation in advancing drug delivery technologies.

While liposomal products approved for drug delivery have been predominantly parenteral, research into gene therapy has yielded liposomal formulations for restoring therapeutic function, demonstrating promising in vivo data [45]. Liposome-mediated gene delivery’s clinical efficacy hinges on the availability of clinically relevant vectors and robust, efficient methods for their delivery to the target cellular site [46,47]. Achieving an optimal balance between the molecular interactions of hydrophilicity and hydrophobicity is essential for effectively transferring genes to the target cells using liposome vectors [45,48]. Local gene delivery at sites of tumor metastases is anticipated to mitigate systemic side effects [49].

Conventional liposomes are an insufficient transfection technology because encapsulating DNA into them presents technical difficulties because of the size of the plasmid [50]. As an alternative, a technology based on cationic lipids and PE was developed in the late 1980s to neutralize the negative charge of plasmids, facilitating their more efficient capture and delivery into cells [47]. 

Because DNA is polyanionic, cationic lipids are usually used for gene delivery, whereas anionic liposomes are mostly used for other therapeutic macromolecule deliveries [51]. When cationic lipid solution is combined with neutral helper lipid solution, DNA may create a positively charged compound called a lipoplex [51,52]. Since it makes it relatively easy to entrap DNA or RNA in a liposome and facilitates efficient gene delivery to cells, DOTMA—one of the first synthesized and commercially available cationic lipids for gene delivery—has greatly increased the potential of nonviral agents for gene therapy [47,51,53]

Liposomes serve as versatile and adaptable nanocarriers for drug delivery, offering benefits such as enhanced biocompatibility and drug isolation. Despite their successes in cancer immunotherapies, challenges like large particle size and rapid immune system clearance persist. Further research is needed to optimize liposomal formulations and explore their integration with emerging technologies like repRNA for groundbreaking advancements in drug delivery and therapeutic applications.

#### 2.1.2. Polymeric Nanoparticle

Polymeric nanoparticles (PNPs) are compounds trapped or adsorbed onto the surface of a polymeric core, and they exhibit variations in characteristics such as concentration, composition, size, shape, and surface properties [54]. The polymeric mass, also known as the nanosphere, entraps the drug within its core or adsorbs it onto its surface [55]. The study and application of this compound are continually expanding, with the most prevalent use being in gene therapy to combat current disorders [56].

The significant potential of this drug delivery type lies in the versatility of the polymers used, making them well-suited for meeting the requirements of various drug delivery systems [57]. Among their advantages, the use of PNPs helps target specific desired sites of action, reducing systemic concentrations and minimizing side effects [57]. Thus, the development of vaccines, such as those for SARS-COV-2, using PNPs in conjunction with self-amplifying RNA, stands as a promising example applied in recent years [58].

Two types of polymers, natural or synthetic, can formulate polymeric nanoparticles based on the therapy’s objectives, administration route, or polymer nature [59]. Natural polymers commonly selected include sodium alginate, albumin, chitosan, and gelatin, while synthetic options encompass polylactides, poly lactide co-glycolides (PLGA), polyglycosides (PGA), polyanhydrides, poly orthoesters (POE), and polycaprolactone (PCL) [55]. 

Polymer-based nanosystems comprise a range of structures, such as hydrogels, polymer micelles, polymerosomes, and polyplexes—which are complexes of cationic polymers and nucleic acids [60,61]. Polyplexes are polymeric systems in which the negatively charged nucleic acids and the cationic groups of the polymer interact electrostatically to include the condensed and/or complexed gene or siRNA [62,63]. The synthesis of nanoparticles with the nucleic acid payload in a compressed and protected state is the outcome of polyplex formation, which is dependent on the entropy-driven ionic interaction between the polyanionic nucleic acid and the multivalent cationic polymers [61].

However, it is important to note that polymeric nanoparticles offer several significant benefits, including the ability to provide controlled release to the target region, stabilize labile molecules (such as proteins), and allow surface modification with ligands for discreet and targeted medication administration [64]. These advantages make them an adjustable and promising option in the field of drug delivery.

Nonetheless, the limitations of polymeric nanoparticles include potential toxic degradation, the aggregation of toxic monomers, residual material attachment, and the possibility of a toxic degradation process within such systems [65]. It is worth noting that using nanoparticles for drug delivery may carry risks due to the increased reactivity and potential toxicity associated with smaller particle sizes [66]. However, various factors, including nanoparticle shape, chemical composition, hydrophobicity/hydrophilicity, and surface charge, may also influence these behaviors [66]. As such, careful consideration and research are essential to harness the full potential of polymeric nanoparticles while mitigating associated risks.

Polymeric nanoparticles offer diverse drug delivery options, targeting specific sites and minimizing side effects. While advantageous in controlled release and stability of labile molecules, they pose risks such as toxic degradation and potential toxicity linked to their small size. Careful research is vital to harness their potential and mitigate associated risks.

#### 2.1.3. Magnetic Nanoparticle

This type of nanoparticle (NP) has garnered extensive research attention and investigation, resulting in its expanded applications [67]. Magnetic nanoparticles (MNPs) have received considerable recognition, primarily for their role in magnetic resonance therapy and magnetic hyperthermia. They offer advantages such as low production costs, high physicochemical stability, acceptable biocompatibility, and biodegradability [68].

Particles possessing both mass and electric charges exhibit magnetic phenomena. This group of particles includes protons, electrons, and holes, as well as positive and negative ions [69]. The functional principle of magnetic nanoparticles centers on their response to magnetic field variations over time, leading to energy transfer from the magnetic field to these nanoparticles [70]. 

Nanoparticles acquire magnetic properties in the presence of an external magnet; however, they revert to their nonmagnetic state once the external magnet is removed [69]. This characteristic prevents the particles from exhibiting magnetic behavior when no external field is applied. This phenomenon induces significant heating and efficient heat transfer, making them suitable for use as chemotherapeutic or radiotherapeutic agents for cancer cell destruction [71]. Moreover, this alternative has proven to be effective without the side effects associated with conventional cancer treatments [72].

Beyond their role in tumor catabolism via hyperthermia, magnetic nanoparticles find applications in various fields [73]. In biomedicine, they facilitate drug or radioisotope transport, serve as magnetic separators for labeled cells, and support various diagnostic mechanisms [74]. This technology can also play a crucial role in sensitive clinical diagnostics for viral RNA, such as SARS-CoV-2, achieved through the synthesis of aminoesters with carboxyl group-coated magnetic nanoparticles and the development of viral RNA extraction methods [75]. 

However, it is important to note that magnetic nanoparticles may have limitations in certain areas, like the environmental industry, due to their high cost and complex operational modifications [76].

The application of an external magnetic field organizes MNPs into a two-dimensional space, imposing a limitation on their concentration within a three-dimensional region for drug administration [77]. Furthermore, removing the external magnetic field makes it challenging to retain the magnetic particles within the targeted organ. Patients cannot experience continuous exposure to an external magnetic field, thereby limiting treatment efficacy, which depends on the frequency, strength, and duration of magnetic field exposure [77]. Despite these challenges, magnetic nanoparticles remain a promising technology with diverse applications, especially in the field of biomedicine.

Magnetic nanoparticles have garnered extensive research attention for their expanded applications in magnetic resonance therapy and magnetic hyperthermia. While they offer advantages such as biocompatibility and stability, challenges remain regarding complex operational modifications and the retention of magnetic properties post removal of the external magnetic field. Despite these challenges, MNPs continue to hold promise for significant advancements in the field of biomedicine, necessitating further research and the enhancement of their applications.

#### 2.1.4. Inorganic Nanoparticles

Nanoshells, also referred to as core shells, consist of cores made of one material encased by an outer shell or coating [78]. Nanoshells typically comprise a dielectric core surrounded by a thin metallic shell, often made of gold, with a thickness ranging from 1 to 20 nm [79]. They can be divided into two main types: oxide nanoshells, originating from oxide core shell particles, including hollow nanoshells, and metal nanoshells, formed using dielectric cores and metallic shells, like silver and gold nanoshells [80]. A common method for producing nanoshells involves using silica as the core material and applying a layer of another substance to attach gold particles to the outer surface, forming the shell [81]. 

According to Ahmadi and Arami (2014) nanoshells have demonstrated significant potential in the field of biomedicine due to their stability, biocompatibility, photoluminescence, tunability, safety, and bioavailability. These applications include gene delivery, drug delivery, targeted therapy, tissue welding, and cancer imaging, among others. However, nanoshells that are conjugated with various molecules, such as antibodies, oligonucleotides, fluorophores, targeting ligands, therapeutic agents, polymers, and radioisotopes, offer enhanced efficacy for diagnostic and therapeutic purposes [82].

The purpose of creating nanoshells is to modify them to absorb light at medically advantageous wavelengths [78]. Nanoshells represent a recent addition to the realm of nanotechnology applications, offering tunable optical properties and the capability to easily conjugate with biomolecules [83]. They offer advantages such as resistance to heat and chemical denaturation, facilitating interactions with medications [84].

They also provide a photothermal effect, facilitating a synergistic combination of chemo–photothermal therapy [85]. The ability to set optical resonance allows for the application of photothermal therapy at various wavelengths of interest, potentially enhancing the mRNA transcription of genes responsible for inducing mitochondrial biogenesis, including sirtuin 1 [86]. This capability has significant implications for fields like muscle tissue engineering, regenerative medicine, and bionics [86]. 

Researchers have used these nanoshell types to eliminate cancer cells in animal models. When injected into a tumor and exposed to radiation, nanoshells heat up sufficiently to destroy the tumor cells [78]. Supporting this, studies have shown that the use of nanoparticles results in higher cancer cell lethality compared to using infrared irradiation alone [87]. However, it is important to note that due to their high surface energy, metal nanoshells can be prone to deactivation, and may exhibit a degree of instability [88].

Nanoshells exhibit great potential in biomedicine due to their stability and tunability, enabling applications in gene and drug delivery, targeted therapy, and cancer imaging. However, their high surface energy may lead to deactivation and instability, necessitating further research for addressing these concerns and fully unlocking their potential in biomedical applications.

#### 2.1.5. Solid Lipid Nanoparticles (SLNs)

Originally known as lipospheres, solid lipid nanoparticles (SLNs) have gained recognition as promising pharmaceutical carriers for controlled drug delivery since 1990, particularly for poorly soluble and bioavailable substances [89,90]. These nanoparticles consist of a lipid matrix along with surfactants and co-surfactants, essential components that provide both stability and ligand properties, resulting in aqueous colloidal dispersions [91]. Lipid nanoparticles (LNPs) consist of a lipid bilayer that encases the RNA, often including additional components such as polyethylene glycol (PEG) and targeting ligands [92]. These components play a crucial role in enhancing stability, extending circulation duration, and enabling targeted delivery to specific cells or tissues [92]. The particle size, which can vary from 10 to 1000 nm, is determined by the chosen preparation method [93].

SLNs exhibit remarkable versatility, as they can encapsulate a wide range of substances, including drugs, vitamins, chemicals, and various xenobiotics [94]. Moreover, they possess the unique capability to transport both anticancer drugs and genes [94]. Comprised mainly of mono-, di-, and triglycerides or fatty acids, these lipid matrices remain in the solid state at typical body temperatures [95].

The choice of lipids in SLN preparation significantly influences their capacity to transport genes, with various lipid substances, such as glyceryl behenate and behenic acid, having been employed as solid lipid matrices [91]. 

This use of solid lipids is particularly advantageous, reducing drug mobility within the lipid matrix and preventing particle coalescence, thereby enhancing stability [96]. Furthermore, the application of SLNs in RNA delivery has not adversely affected RNA functionality, showing promise for both drug and gene delivery [97]. 

Ionizable lipids are considered crucial components of LNP-based RNA therapeutics because they are positively charged at a low pH to enhance the encapsulation of negatively charged RNA, and the charge becomes less positive or almost neutral at physiological pH [98]. A non-bilayer structure is formed by the interaction of these positively charged ionizable lipids with the negatively charged inner endosome membrane [99]

As the demand for advanced disease therapies grows, SLNs with complex internal structures provide improved stability and offer advantages over conventional biopharmaceuticals and other nanoscale carriers [94,100]. These advantages include minimal biotoxicity due to the biocompatible and biodegradable nature of the lipids used [101]. They can be produced without the need for organic solvents and provide excellent structural stability [101,102]. SLNs are versatile, allowing for both drug targeting and controlled drug release, and the incorporation of active compounds enhances their stability [101,103]. Additionally, SLNs are capable of encapsulating lipophilic and hydrophilic drugs, making them adaptable to various therapeutic applications [101,104]. Furthermore, producing SLNs on a large scale is simple, and they are sterilizable [101,105].

Despite these numerous advantages, SLNs also come with potential limitations, including relatively low drug-loading capacity depending on the environment and the physicochemical composition of the active ingredients, especially hydrophilic molecules [101,106]. Additionally, the polymorphic nature of the medication may lead to drug leakage from the carrier, particularly during storage, when phase transitions are likely to occur [101,107]. Another concern is the relatively high-water content in SLN dispersions, which can range from 70% to 99.9% [101,108].

While the absence of SLNs in clinical applications may be attributed to a lack of rational cost–benefit analysis, they still hold promise for the pharmacokinetic profiling of encapsulated drugs [109].

SLNs have shown promise in controlled drug delivery due to their enhanced stability and biocompatibility, but their drawbacks include low drug-loading capacity for hydrophilic molecules, potential drug leakage during storage, and high water content in SLN dispersions. Further research is needed to address these limitations and fully integrate SLNs into clinical practice.

### 2.2. Nanotoxicology Challenges and Opportunities

Nanotoxicology, while an expanding field, still has significant gaps, with existing research primarily focused on acute toxicity, necessitating an in-depth exploration of long-term toxicity and chronic exposure effects [110]. The comprehensive evaluation of the safety and toxicity of nanodrug delivery systems remains challenging due to various potential factors contributing to nanotoxicity and the potential for unpredictable interactions with biological systems [110,111]. 

The integration of validated analytic methodologies and well-designed experiments can elucidate toxicity mechanisms, ensuring the safe utilization of nanoformulations in therapeutic and diagnostic applications [112,113]. Advancements in nanobiotechnology, material synthesis, and computer simulation studies offer promising prospects for engineering nanoformulations that prioritize both safety and efficacy, thereby mitigating potential toxicities [112]. Notably, regulatory bodies such as EMA and FDA have established guidelines to oversee the application of nanoformulations in medicine [110]. 

Consequently, further toxicological investigations are imperative to enable an in-depth understanding of nanoparticle behavior, allowing for the enhancement of their properties to minimize toxicity and maximize therapeutic efficacy [111,114]. Studies have documented various toxic effects associated with different nanoparticles, including increased oxidative stress and potential intracellular infiltration by metal-based nanoparticles, as well as observed hepatotoxicity and nephrotoxicity from protein-based nanoparticles [111,115,116]. 

## 3. Virus-Based Delivery System

Viral vectors, which make use of viruses’ ability to transfer genetic information into host cells, have shown promise in gene therapy systems [117]. Numerous altered viral vector genomes have been created, such as lentiviruses, herpes viruses, adenoviruses, adeno-associated viruses, retroviruses, human foamy viruses (HFVs), and herpes viruses [118,119] These modified genomes have certain limitations, including immunogenicity, toxin production, insertional mutagenesis, and constraints in transgenic capacity size [120,121]. Recent advancements have led to the design of viral vectors with specific receptors for the transfer of transgenes to non-natural target cells, a concept known as retargeting [120].

Retroviral vectors are commonly used for gene delivery in both somatic and germline gene therapies due to their ability to transfect dividing cells by passing through the nuclear pores of mitotic cells [122]. This feature renders retroviruses suitable candidates for in situ treatments, distinguishing them from adenoviral and lentiviral vectors [120]. These viruses integrate with the host genome, allowing the production of viral proteins, such as gag, pol, and env, that are extracted during gene delivery [123]. The coordinated design of packaging cell lines and retroviral expression vectors is the foundation of retroviral gene transfer technology, which makes it easier to package recombinant retroviral RNAs into infectious, replication-incompetent particles [124]. 

Lentiviruses, a subclass of retroviruses, have emerged as significant gene delivery vectors, distinguished by their unique capacity to naturally integrate with nondividing cells [125]. This characteristic sets them apart from other retroviruses, which can solely infect dividing cells [126]. The strong tropism of lentiviruses for neural stem cells has rendered them invaluable for ex vivo gene transfer in the central nervous system, exhibiting no significant immune responses or unwanted side effects [127,128]. These vectors offer several advantages, including the high-efficiency infection of both dividing and nondividing cells, long-term stable expression of a transgene, low immunogenicity, and the ability to accommodate larger transgenes [120,129]. Due to their ability to integrate the transgene into the host genome and enable long-term expression, lentiviral vectors have remained a compelling option for clinical gene delivery [130].

Adenoviral vectors, derived from various species and boasting over 100 different serotypes [131], exhibit a versatile nature that allows them to be employed for transferring genetic material into both dividing and non-dividing cells [128]. Notably, adenoviruses such as type 2 and 5 possess low host specificity, enabling their utilization in gene delivery across a wide range of tissues [120]. Large DNA particles up to 38 kb may be transported by these vectors, but unlike retroviruses, they do not integrate into the host genome, causing comparatively short-term gene activation [120,132]. Adenoviral vectors exist in different generations, with the initial generation involving the deletion of the E1 gene, which might trigger acute and chronic immune responses [130,133]. Subsequent generations have seen the removal of the E2 and E4 genes to mitigate the immune response [130].

These systems benefit from the structural protection of the virus, preventing DNA degradation via the lysosome [117]. RNA-based gene delivery has also gained attention, with systems such as oncoretroviral vectors and lentiviral vectors, offering transient gene expression [117,134]. Although viral gene delivery technologies provide therapeutic and continuous gene expression, there are still issues with manufacturing, immunogenicity, toxicity, and the requirement for extensive tuning [135]. Researchers continue to explore the optimal design of viral vectors, considering the specific virus type to be used [135,136].

## 4. Advantages and Challenges of Replicon RNA Therapy

The application of nucleic acid-based therapy offers promise in addressing diseases that traditional treatments struggle to manage [137,138]. Self-replicating RNA, known as replicon RNA (RepRNA), represents a novel frontier in gene therapy [137,139]. These RepRNA molecules, derived from RNA viruses with specific gene deletions, can amplify all encoded genetic information, resulting in high antigen expression levels [137]. This sets RepRNA apart from plasmid DNA-based vaccines, which depend on delivering initial genetic information to the nucleus [139]. 

mRNA therapy, another breakthrough, leverages nanoparticles as intracellular delivery systems for normal genes [138,140]. These synthetically manipulated mRNAs can replace missing or defective genes and express transient proteins to correct genetic disorders and promote beneficial mechanisms or pathways, ultimately aiming to cure or treat pathologies [16,141]. 

repRNA’s favorable characteristics are evident in its therapeutic potential [139]. RepRNA-based vaccines have proven safe and potent in clinical trials against various infectious diseases and cancers [142]. RepRNA serves as a template for increased RNA molecule translation, leading to more rounds of antigen production [143]. Unlike DNA, which requires entry into the cell nucleus, both mRNA and RepRNA perform protein translation in the cytoplasm, reducing the risk of gene interactions [139].

**Figure 1:**
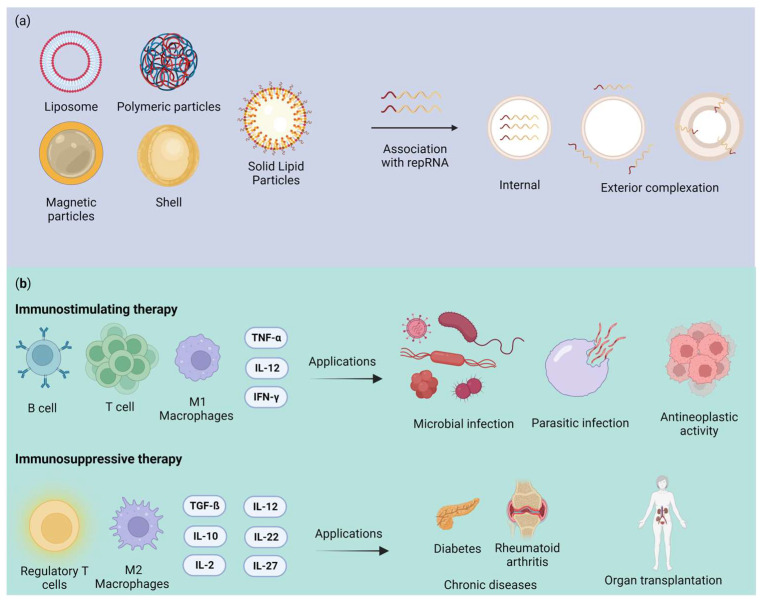
Overview of the use of nanoformulations associated with repRNA: (**a**) examples of nanoformulations and forms of association with repRNA; (**b**) immunomodulatory potential of nanoparticles associated with repRNA and therapeutic application, either by immunostimulation or immunodepression. The information contained in this illustration was based on the work of Blakney [144] and Feng et al. [145]. Created with BioRender.com (accessed on 2 October 2023).

In the comprehensive exploration of nanoformulations integrated with repRNA technology, a multifaceted approach unfolds. The synergy between various nanoformulations and repRNA is visually depicted in Figure 1a, illustrating the diverse forms of association that empower this innovative therapeutic approach. From liposomes to polymeric nanoparticles, this mix enhances the pharmacological potential of repRNA. These nanoformulations also delve into the realm of immunomodulation, as illustrated in Figure 1b. They possess the remarkable capacity to either stimulate or suppress the immune response, opening the door to a range of therapeutic applications. Whether due to their potential for immunostimulation to combat infections or employing them for immunodepression in autoimmune disorders, the versatility of nanoformulations associated with repRNA holds immense promise in shaping the future of medical treatments [144,145].

The RepRNA production system is based on the loading of nanoparticles with RepRNA, followed by the efficient uptake of this formulation by target cells. These nanoparticles have a protective role against RNAses and degradation processes, ensuring the integrity of the encapsulated RNA [146]. Once inside the cellular cytoplasm, the nanoparticles underwent controlled disintegration, facilitating the amplification of RNA and a subsequent increase in the number of copies and protein expression [146]. This orchestrated process ultimately led to the presentation of RNA-derived antigens to the major histocompatibility complex (MHC), eliciting a robust immune response and highlighting the potential of our nanoparticle-based RNA delivery system.

Furthermore, RepRNA production is considered simpler and more cost-effective than mRNA production, as it generates superior immune responses with significantly lower RNA doses due to the presence of RNA replicons [139]. However, despite these advantages, RepRNA therapy still faces major challenges [1,16]. 

The sizeable nature of RepRNA presents a significant obstacle to efficient cytosolic delivery, particularly when compared to smaller conventional mRNA [8]. The significant challenges associated with large and intricate RepRNA vaccines primarily revolve around their vulnerability to ribonucleases (RNases) and the ineffective translation process within dendritic cells (DCs) [7]. Consequently, one of the primary hurdles to expanding the extensive potential of RepRNA vaccines lies in the imperative requirement for safe and effective in vivo delivery strategies [8]. These limitations underscore the critical need for innovative strategies to mitigate the impact of these obstacles and enhance the overall efficacy of RepRNA vaccines.

One significant challenge lies in the delivery of RNA, given its negative charge and large molecular size, making it difficult to traverse the anionic lipid bilayer of cells [137]. Additionally, its single-stranded nature renders it fragile and susceptible to degradation by RNase enzymes, hindering its intracellular entry and limiting its applications in gene therapy [147]. Recent scientific and technological advances have made strides in overcoming these RNA-associated obstacles, leading to the development of efficient delivery systems that protect RNA from degradation, break through cell membranes, and deliver RNA into the cytoplasm [148]. 

Another challenge is the route of administration, primarily intravenous (i.v.) delivery, which targets highly vascularized organs like the liver or spleen to achieve therapeutic protein expression in a sufficient number of transfected cells [149]. However, this approach requires prolonged administration times and results in high treatment costs, often necessitating hospitalization or monitoring in infusion centers. Therefore, researchers have increasingly explored subcutaneous or intramuscular applications to address these limitations [142].

In recent years, RepRNA has gained attention as a potential therapy for COVID-19, and its success has stimulated interest in its application for various other pathologies (Table 2).

Notably, McCullough and collaborators (2014) laid the foundation by demonstrating the transport of self-amplifying, cap-independent replicon RNA to dendritic cells (DCs), resulting in increased RepRNA translation and the activation of the immune response [156].

Garcia et al. (2018) conducted a study to evaluate the impact of macrophage migration inhibitory factor (PMIF) immunoneutralization on host responses. Their research revealed remarkable advancements, including the enhanced control of liver and blood-stage *Plasmodium* infection and complete protection against reinfection, through a novel RNA replicon-based vaccine. A cationic nanoemulsion was prepared by combining squalene, DOTAP, and sorbitan trioleate, and it was characterized for particle size, RNAse protection, and endotoxin, as previously described [150,157]. The complexation process was allowed to continue for at least 30 min before immunization [150]. Intriguingly, the study observed a delay in the onset of blood-stage patency following sporozoite infection [150]. Furthermore, vaccination against PMIF resulted in notable improvements in T follicular helper (Tfh) cell and germinal center responses, a reduction in the expression of Th1-associated inflammatory markers such as TNF-α, IL-12, and IFN-γ during the blood-stage infection, elevated anti-*Plasmodium* antibody levels, and the enhanced differentiation of antigen-experienced memory CD4 T cells and liver-resident CD8 T cells [150].

To facilitate the delivery of an alphavirus replicon encoding the highly neutralizing human monoclonal antibody (mAb) ZIKV-117, Erasmus et al. (2020) engineered a nanostructured lipid carrier (NLC). These NLC nanoparticles exhibit exceptional colloidal stability attributed to their hybrid core composed of liquid squalene and solid glyceryl trimyristate (Dynasan 114) [158]. Their studies have demonstrated robust protection, both as pre-exposure prophylaxis and post-exposure therapy, following alphavirus-driven expression of ZIKV-117 mRNA delivered via intramuscular injection (IM) [151]. The nanoparticle formulation was manufactured using a specific composition [151,158]. The oil phase was composed of squalene and glyceryl trimyristate, along with a non-ionic sorbitan ester surfactant, and the cationic lipid DOTAP. The aqueous phase consisted of a 10 mM sodium citrate trihydrate buffer containing the non-ionic PEGylated surfactant Tween 80. Additionally, the researchers successfully optimized the expression of human IgG from repRNA, enabling the production of protective levels in mice [151].

Furthermore, the same research group unveiled the LION/repRNA-CoV2S vaccine, exhibiting substantial neutralization of SARS-CoV-2 and inducing potent S-specific T cell responses in vaccinated mice and pigtail macaques [142]. According to Erasmus et al. (2020) LION is a highly stable cationic squalene emulsion containing 15 nm superparamagnetic iron oxide (Fe_3_O_4_) nanoparticles (SPIO) incorporated in the hydrophobic oil phase. This vaccine’s ability to stimulate memory T cell responses specific to SARS-CoV-2 in macaques may contribute to long-term protection against and recovery from SARS-CoV-2 infection [142].

Recent findings by Leventhal et al. (2022) have significantly improved the understanding of how vaccines against the Crimean–Congo hemorrhagic fever virus (CCHFV) provide protection. Vaccine RNA was synthesized in vitro and subsequently complexed to cationic nanocarrier, following established protocols as described previously by Erasmus et al. (2020). Their research has shed light on the involvement of humoral and cellular immunity in repRNA-mediated protection. Importantly, administering RNA through straightforward intramuscular vaccinations, particularly with the use of cationic emulsions, has emerged as an essential strategy for enhancing RNA stability and transport [152].

In the study conducted by Fotoran et al., tests were managed on a self-amplifying repRNA (repRNA) vaccine formulation, which was based on the SP6 Venezuelan equine encephalitis (VEE) vector incorporated into cationic liposomes. They generated three vaccines that encoded two reporter genes (GFP and nanoLuc) and the *Plasmodium falciparum* reticulocyte binding protein homologue 5 (PfRH5). The liposome–replicon complexes exhibited high transfection efficiencies and elicited antibodies capable of inhibiting the growth of the parasite in vitro [153].

To find out how mannans of different lengths (from mono to tetrasaccharide) affected the antibody response of a model repRNA replicon that encodes the respiratory syncytial virus fusion F protein, a new set of LNPs with changed surfaces was created by Goswami et al. (2021). As the mannose chain length increased, the vaccination priming response showed a steady improvement; nevertheless, the response to the booster dose peaked at a length greater than the disaccharide. Mannosylated lipid nanoparticles (MLNPs) were shown to exhibit higher amounts of IgG1 and IgG2a in comparison to LNPs. The potential of mannosylated samRNA LNPs for intramuscular and intradermal distribution is confirmed by this study [154].

Chahala et al. (2016) created a single-dose, completely synthetic, adjuvant-free dendrimer nanoparticle vaccination platform with a fast response, and encapsulated mRNA replicons that encode antigens. Protective immunity against a wide range of deadly pathogen threats, including as the Ebola virus, *Toxoplasma gondii*, and H1N1 influenza, can be produced via this mechanism [155]. The vaccine possesses the ability to induce both CD8+ T-cell and antibody responses and may be designed with various antigen-expressing replicons [155].

## 5. Advances and Perspectives

Various RNA-based therapies have emerged, including RNA-directed drugs involving chemically modified oligonucleotides, which represent a significant advancement in the field of RNA therapeutics [159]. The extensive range of diseases currently undergoing clinical trials highlights the potential of nanoparticles, especially lipid nanoparticles (LNPs), in the realm of RNA-based therapies [160]. Additionally, coupled nanoformulations have exhibited promise in various treatments (Table 3). 

Massad-Massade et al. (2018) synthesized siRNA-SQ (squalene) and siRNA-SOLA (solanesol) and achieved the production of effective siRNA-nanoparticles using an innovative method that easily adapts to pharmaceutical development. They utilized nanoprecipitation in acetone/water to create the nanoparticles [161].

The simplicity and adaptability of the researchers’ proposed methodology, along with the increased synthesis yield, provide a novel approach for targeting overexpressed genes using siRNA–polyisoprenoid-conjugated nanoparticles, thereby reducing the risk of malignancies or genetic disorders [161,163,164,165]. 

The study conducted by Guo et al. in 2020 showed that intravenous administrations of RNA-paclitaxel nanoparticles, integrated with precise cancer-targeting ligands, efficiently addressed the solubility challenges of hydrophobic drugs in patient care. Notably, this strategy resulted in a significant suppression of breast cancer growth in mice, accompanied by negligible levels of toxicity and immune responses [162].

Furthermore, Song et al. (2020) designed mesoporous silica nanoparticles (MSN) loaded with myricetin (Myr) and multidrug resistance protein (MRP-1) siRNA and modified with folic acid (FA) to enhance therapeutic efficacy for non-small cell lung cancer (NSCLC) treatment. These nanoparticles exhibited sustained Myr release and improved uptake by lung cancer cells compared to non-targeted ones. The in vitro drug release data confirmed sustained release in FA-conjugated MSN with Myr and MRP-1 nanoparticles [166]. Treatment involving FA-conjugated MSN with Myr and MRP-1 significantly reduced the viability of lung cancer cell lines (A549 and NCI-H1299) and inhibited colony formation [166].

The potential of mRNA technology in various applications, including cancer immunotherapy and infectious disease vaccination, is evident, but its success depends on improving stability and reducing immunogenicity [2]. Moreover, due to electrostatic repulsion by cell membranes and vulnerability to RNases, mRNA requires protection and efficient transport to target cells. Over the years, researchers have harnessed lipid vesicles to safeguard and deliver RNA molecules, with advancements incorporating structural lipids to mimic cell membranes and shield the positive charge [171].

Recent approvals of RNA-based technologies, including Patisiran (Onpattro^®^), Givoriran from Alnylam, and the emergency-use mRNA-LNP vaccine for COVID-19 (Comirnaty^®^), underscore the safety and efficacy of LNP-based nucleic acid delivery [160,171,172].

Patisiran, an innovative medicine, uses a liposomal siRNA to selectively target transthyretin (TTR), a plasma transport protein for retinol-binding protein and thyroid hormone [167]. This RNA interference (RNAi) therapy treats polyneuropathy associated with transthyretin amyloidosis (ATTR) [168]. Patisiran’s therapeutic component consists of a novel siRNA formulated as a lipid nanoparticle, which effectively reduces TTR accumulation in tissues, leading to improvements in cardiac and neuropathic function [167,168].

Givosiran, developed by Alnylam Pharmaceuticals in Cambridge, Massachusetts, is a highly selective siRNA designed to reduce the occurrence of recurrent episodes in conditions like acute intermittent porphyria (AIP) by downregulating hepatic delta-aminolevulinic acid synthase 1 (ALAS1) enzyme expression [169,170]. This siRNA, which specifically targets the messenger RNA (mRNA) encoding ALAS1, effectively reduces induced ALAS1 levels, preventing the accumulation of potentially harmful porphyrin precursors such as porphobilinogen (PBG) and delta-aminolevulinic acid (ALA) [169,170]. Givosiran demonstrates selectivity for hepatocytes due to its linkage to N-acetylgalactosamine (GalNac), facilitating its uptake through asialoglycoprotein receptors (ASGPR) [169]. While Givosiran demonstrates efficacy in reducing recurrent episodes, it also has potential adverse effects, including hepatic, renal, and hyperhomocysteinemia risks [169].

The durability of mRNA-LNP vaccines depends on their chemical composition, with various mRNA vaccine candidates created during the development of vaccines targeting SARS-CoV-2 [173]. Among these candidates, three “conventional” mRNA vaccines, all encoding the entire S protein, have advanced to advanced clinical development stages. These vaccines are the Moderna mRNA-1273 vaccine, the BioNTech/Pfizer BNT162b2/Comirnaty vaccine, and the CureVac CVnCoV vaccine [173].

Nonetheless, while LNPs offer a highly customizable platform for nucleic acid delivery, particularly in mRNA vaccines, and exhibit potential in therapeutics for rare diseases and cancers, it is important to note that there is no one-size-fits-all solution for all diseases, necessitating ongoing optimization efforts [171]. Amid heightened public interest in mRNA vaccines, scientists have dedicated a wealth of scientific research to design and refine this technology, ushering in a new era in nanomedicine [160,171,172,174].

## 6. Conclusions

The rapid progress in the field of RNA-based therapeutics, driven by the resounding success of mRNA technology and the urgency brought about by the COVID-19 pandemic, has illuminated a promising path for repRNA technology. The recent approvals of mRNA-based products have not only demonstrated the regulatory acceptance, but also highlighted the immense potential of the RNA platform in modern medicine. As we find ourselves at the crossroads of innovation in nanomedicine, the potential of repRNA technology as a versatile tool against a myriad of diseases becomes evident. Its distinctive capacity for high-level antigen expression, simplified production processes, and the potential for lower therapeutic doses have positioned it as a formidable contender for the future of medical therapies. However, to fully realize its potential and ensure its place as a transformative technology, we must address several key challenges and consider specific future directions.

Future research should prioritize increasing the stability of repRNA to mitigate the different degradation possibilities, making its application more multifaceted. Improving resistance to degradation is pivotal for ensuring the sustained therapeutic effect of repRNA-based treatments. Moreover, to unlock the full potential of repRNA, researchers must continue to explore innovative methods for efficient and targeted delivery, overcoming obstacles in delivering repRNA to specific cells or tissues. As repRNA therapies advance, understanding and mitigating their immunogenicity remains a crucial area of study. A focus on minimizing immune responses and side effects is vital to ensure patient safety and treatment effectiveness. Beyond infectious diseases, repRNA holds promise in addressing genetic disorders, cancer, and chronic illnesses. Future research and development efforts should expand its applications. To facilitate the integration of repRNA into mainstream medical practice, a robust and clear regulatory framework must be established. This framework should encourage innovation while ensuring patient safety and equitable access. The momentum in RNA-based therapeutics is undeniable, and with sustained research and innovation, repRNA stands to become one of the most transformative medical technologies of our time. It offers the promise of more effective treatments and potential cures across a range of illnesses, making the future of medicine brighter and more hopeful.

## Figures and Tables

**Table 1:** Nanoformulations and their main components approved for therapeutic use by FDA and regulatory agencies.

Classification	Name (Trade Tame)	Main Component	Delivery Route	Indication	Approval (Year)
Liposome	AmBisome	Liposomal amphotericin B	Intravenous	Fungal/protozoal infections	FDA (1997) EMA (2006) ANVISA (1997)
	Doxil/ Caelyx	Liposomal doxorubicin	Intravenous	Antineoplastic agents (ovarian and breast cancer; multiple myeloma; Karposi’s Sarcoma)	FDA (1995, 2005, 2008) EMA (1996) ANVISA (2011)
	Myocet (Myoce liposomal)	Liposomal doxorubicin	Intravenous	Antineoplastic agents (breast neoplasms)	FDA (2000) EMA (2000)
	Visudyne	Liposomal verteporfin	Intravenous	Ophthalmic agents (myopia; ocular histoplasmosis; macular degeneration, wet age-related)	FDA (2000) ANVISA (2004) EMA (2007)
	Marqibo	Liposomal vincristine	Intravenous	Antineoplastic agents (hematologic malignancies and solid tumors)	FDA (2012)
	Onivyde (Onivyde pegylated liposomal)	Liposomal irinotecan	Intravenous	Antineoplastic agents (pancreatic cancer)	FDA (2015) EMA (2016)
Polymer-based nanoparticles	Eligard	Leuprolide acetate and polymer (PLGH (poly (DL-Lactide-co-glycolide)))	Subcutaneous	Antineoplastic agents (prostate cancer)	FDA (2002) ANVISA (2006)
	Mircera	Methoxy polyethylene glycol-epoetin beta	Subcutaneous/ Intravenous	Anemia associated with chronic kidney disease	FDA (2007) EMA (2007) ANVISA (2008)
	Cimzia	PEGylated antibody fragment (Certolizumab)	Subcutaneous	Anti-inflammatory action (Crohn’s disease; rheumatoid arthritis; psoriatic arthritis; ankylosing spondylitis)	FDA (2008, 2009, 2013) EMA (2009) ANVISA (2017)
	PegIntron	PEGylated IFN alpha-2b protein	Subcutaneous	Immunomodulator (hepatitis C)	FDA (2001) EMA (2000) ANVISA (2011)
Magnetic nanoparticles	NanoTherm	Iron oxide coated with amino silane	Intratumoral injection	Antineoplastic agents (glioblastoma)	FDA (2010) EMA (2013)
	Feraheme	Iron oxide and a polyglucose sorbitol carboxymethyether	Intravenous	Treatment of anemia	FDA (2009) EMA (2012)
Lipid nanoparticle	Patisiran (Onpattro)	Phospholipids, cholesterol, ionizable cationic lipid (DLin-MC3-DMA), and polyethylene glycol-modified lipid	Intravenous	Polyneuropathy	FDA (2018)

**Table 2:** Application of repRNA associated with a nanocarrier for the therapy of pathologies.

Carrier	RNA Replicon	Results	Reference
Cationic nanocarrier	RepRNA PMIF (macrophage migration inhibitory factor and cytokine).	It improved host cellular and humoral immunity against Plasmodium infection in the liver and blood and conferred complete protection against malaria reinfection in murine mice.	[150]
Nanostructured lipid transporters (NLCs)	RepRNA ZIKV-117 mAb.	Rapid protection against Zika virus infection in mice.	[151]
Lipid InOrganic Nanoparticles (LION)	LION/repRNA-CoV2S	LION/repRNA-CoV2S vaccine intramuscularly to mice, a significant amount of anti-SARS-CoV-2 S protein IgG antibody isotypes, resembling a Type 1 T helper cell response, were produced.	[142]
Cationic nanocarrier	RepRNA CCHFV (Crimean–Congo hemorrhagic fever virus) encoding NP (nucleoprotein), GPC (glycoprotein precursor) or both	It provided robust protection against Crimean–Congo hemorrhagic fever virus in lethal mice.	[152]
Cationic liposomes	samPfRH5 replicon (*Plasmodium falciparum* reticulocyte binding protein homologue 5)	The liposome–replicon complexes showed high transfection efficiencies. They elicited antibodies capable of inhibiting the growth of the parasite in vitro	[153]
Mannosylation of lipid nanoparticles (LNPs)	Self-amplifying mRNA encoded an influenza (hemagglutinin)	Compared to LNPs, mannnosylated lipid nanoparticles (MLNPs) showed higher levels of IgG1 and IgG2a.	[154]
Polymeric nanoparticle	Nanoparticle (MDNP)-delivered VEEV replicon RNA encoding the hemagglutinin protein (HA) of an H1N1 influenza virus (A/WSN/33) or the Ebola virus (EBOV) glycoprotein (GP)	The vaccine elicits both CD8+ T-cell and antibody responses and can be created with numerous antigen-expressing replicons.	[155]

**Table 3:** Applications of RNA conjugated with nanoparticles used as therapy for diseases.

Technology	Application	Type of Nanocarrier	References
siRNA-SQ and siRNA-SOLA	Cancers Harboring Fusion Oncogenes	Polyisoprenoid chains	[161]
RNA-paclitaxel	Breast cancer treatment	Lipidic	[162,163,164,165]
MRP-1 siRNA	Non-small cell lung cancer	Mesoporous silica	[166]
ALN-18328 (Patisiran)	Transthyretin amyloidosis (ATTR)	Lipid nanoparticle	[167,168]
GalNAc-siRNA (Givosiran)	Acute hepatic porphyria	Lipid nanoparticle	[169,170]
siRNA-LNP	Vaccine for COVID-19	Lipid nanoparticle	

## Data Availability

Data sharing is not applicable.
